# *HMGA2* expression pattern and *TERT* mutations in tumors of the vulva

**DOI:** 10.3892/or.2015.3882

**Published:** 2015-03-30

**Authors:** ANTONIO AGOSTINI, IOANNIS PANAGOPOULOS, HEGE KILEN ANDERSEN, LENE ELISABETH JOHANNESEN, BEN DAVIDSON, CLAES GÖRAN TROPÉ, SVERRE HEIM, FRANCESCA MICCI

**Affiliations:** 1 Section for Cancer Cytogenetics, Institute for Cancer Genetics and Informatics, The Norwegian Radium Hospital, Oslo University Hospital, Oslo, Norway; 2 Centre for Cancer Biomedicine, University of Oslo, Oslo, Norway; 3 Department of Pathology, The Norwegian Radium Hospital, Oslo University Hospital, Oslo, Norway; 4 Faculty of Medicine, University of Oslo, Oslo, Norway; 5 Department of Gynecology, The Norwegian Radium Hospital, Oslo University Hospital, Oslo, Norway

**Keywords:** malignant melanoma, squamous cell carcinoma, *HMGA2*, *HMGA1*, *IDH1*, *IDH2*, *TERT*, *MGMT*

## Abstract

Malignant tumors of the vulva account for only 5% of cancers of the female genital tract in the USA. The most frequent cancers of the vulva are squamous cell carcinoma (SCC) and malignant melanoma (MM). Little is known about the genetic aberrations carried by these tumors. We report a detailed study of 25 vulva tumors [22 SCC, 2 MM, 1 atypical squamous cell hyperplasia (AH)] analyzed for expression of the high-mobility group AT-hook family member genes *HMGA2* and *HMGA1*, for mutations in the *IDH1*, *IDH2* and *TERT* genes, and for methylation of the *MGMT* promoter. The RT-PCR and immunohistochemistry analyses showed that *HMGA2* was expressed in the great majority of analyzed samples (20 out of 24; SCC as well as MM) but not in the normal controls. *HMGA1*, on the other hand, was expressed in both tumors and normal tissues. Five of the 24 tumors (all SCC) showed the C228T mutation in the *TERT* promoter. Our results showed that *HMGA2* and *TERT* may be of importance in the genesis and/or the progression of tumors of the vulva.

## Introduction

Malignant tumors of the vulva account for 5% of cancers of the female genital tract in the USA (1). Squamous cell carcinomas (SCC) make up 70% of all vulvar cancers. The incidence rate is much higher in older (20:100,000) than in younger (1:100,000) women (1). Little is known about the acquired genomic changes of this type of cancer as only few cases have been cytogenetically and/or molecularly analyzed (2–4). Chromosome and array-based comparative genomic hybridization (CGH) analysis (4) has shown, among other imbalances, loss of chromosomal band 3p14 leading to deregulation of the *FHIT* gene. Expression array analyses also identified a number of other genes whose expression profile was altered. Notable among the downregulated genes were *MAL* (in 2q11), *KRT4* (in 12q13) and *OLFM4* (in 13q14), whereas upregulated genes included *SPRR2G* (in 1q21.3) and *S100A7A* (in 1q21.3). No mutation analyses have so far been performed in this tumor type. Vulva malignant melanoma (MM), the second most common malignancy in this organ or site, is an aggressive cancer carrying poor overall prognosis (5). Only five vulvar MM cases have been cytogenetically characterized; they showed complex karyotypes with no recurrent aberration. No molecular genetic data on vulvar MM cases have been published.

To gain more information on the genetics of vulvar tumors, we analyzed 22 SCC, two MM, and one atypical squamous cell hyperplasia (AH) case for expression of the high-mobility group AT-hook genes *HMGA2* and *HMGA1*. We then searched them for mutations in the isocitrate dehydrogenase 1 (*IDH1*) and 2 (*IDH2*) and telomerase reverse transcriptase (*TERT*) genes, and checked them for the methylation status of the promoter of O^6^-methylguanine-DNA methyltransferase (*MGMT*). Since these genes have all been found mutated, deregulated and/or methylated in various types of cancer (6–9), they seemed to be a reasonable starting point for the characterization of the genetic profile also of vulvar cancers.

## Materials and methods

### Tumor material

The material consisted of fresh samples from 22 SCC, two MM and one AH, all arising in the vulva and surgically removed at The Norwegian Radium Hospital between 1998 and 2009 (Table I). The tumors have been previously characterized for chromosomal aberrations and genomic imbalances (3); a subset of them was also investigated for their expression profile (4).

### DNA and RNA extraction and cDNA synthesis

DNA extraction was performed on 24 samples (no frozen material was available for case 15). The DNA was extracted using the Maxwell 16 extractor (Promega, Madison, WI, USA) and the Maxwell 16 Tissue DNA Purification kit (Promega) according to the manufacturer’s recommendations. RNA was extracted from the 12 samples from which we had sufficient material to extract both DNA and RNA. The RNA was extracted using the miRNeasy kit (Qiagen, Hilden, Germany) and QIAcube (Qiagen). The concentration and purity of both DNA and RNA were measured with the NanoVue spectrophotometer (GE Healthcare, Pittsburgh, PA, USA). One microgram of extracted RNA was reverse-transcribed in a 20 *μ*l reaction volume using the iScript Advanced cDNA Synthesis kit according to the manifacturer’s instructions (Bio-Rad Laboratories, Oslo, Nor way).

### Molecular analyses

All primers used in the PCR reactions are listed in Table II. All PCR reactions were run on a Bio-Rad C100 Thermal Cycler (Bio-Rad Laboratories). Three microliters of the PCR products were stained with GelRed (Biotium, Hayward, CA, USA) and analyzed by electrophoresis through 1.0% agarose gel. The gel was scanned with G-Box (Syngene, Los Altos, CA, USA) and the images were acquired using GeneSnap (Syngene). The remaining 22 *μ*l of the amplified fragments were purified using the QIAquick PCR Purification kit (Qiagen). Direct sequencing was performed using the light run sequencing service of GATC Biotech (http://www.gatc-biotech.com/en/sanger-services/lightrun-sequencing.html). The BLAST (http://blast.ncbi.nlm.nih.gov/blast.cgi) and BLAT (http://genome.ucsc.edu/cgi-bin/hgblat) programs were used for computer analysis of sequence data.

### Reverse transcriptase-polymerase chain reaction (RT-PCR)

cDNA equivalent to 10 ng RNA was amplified using the Takara Premix Ex Taq (Takara-Bio, Europe/SAS, Saint-Germain-en-Laye, France). The primer combination HMGA2-846F1 and HMGA2-1021R1 was used to amplify the region between exons 1 and 3, whereas the primer combination HMGA2-846F1 and HMGA2-1112R1 was used for exons 1 to 5. Expression of the housekeeping gene *ABL1* was monitored as the internal control. The PCR cycling program for both *HMGA2* and *ABL1* was as follows: 30 sec at 94°C, followed by 35 cycles of 7 sec at 98°C and 2 min at 68°C and a final step at 68°C for 5 min. The RT-PCR products were analyzed by electrophoresis. The primers HMGA1-284F1 and HMGA1-648R1 were used to amplify the *HMGA1* transcript. The PCR cycling program for *HMGA1* was as follows: 30 sec at 94°C followed by 35 cycles of 7 sec at 98°C, 30 sec at 55°C, 60 sec at 72°C and a final extension for 2 min at 72°C.

### 3′ Rapid amplification of cDNA ends - PCR (3′RACE-PCR)

For 3′-RACE-PCR, 100 ng of total RNA were reverse-transcribed in a 20 *μ*l reaction volume with A3RNV-RACE as a primer and using the iScript Select cDNA Synthesis kit according to the manufacturer’s instructions (Bio-Rad Laboratories). One microliter was used as a template and amplified using the outer primer combination HMGA2-846F1/A3R-1New. One microliter of the amplified products was used as template in nested PCR with the primers HMGA2-982F1 and A3R3. For both PCRs the 25 *μ*l reaction volume contained 12.5 *μ*l of Premix Ex Taq (Takara-Bio), template and 0.4 *μ*M of each of the forward and reverse primers. PCR cycling consisted of an initial step of denaturation at 94°C for 30 sec followed by 35 cycles of 7 sec at 98°C, 30 sec at 55°C, 90 sec at 72°C and a final extension for 5 min at 72°C. The PCR products were analyzed by electrophoresis, purified and sequenced.

### Polymerase chain reaction (PCR)

#### IDH1 and IDH2

DNA was first amplified in a 25 *μ*l reaction volume using Takara Premix Ex Taq and 1 *μ*l of the primer combination IDH1-rs1-86F and IDH1-rs1-321R for *IDH1,* and IDH2-rs12-42F and IDH2-rs12-315R for *IDH2.* The thermal cycling for *IDH1* included an initial step at 94°C for 30 sec, followed by 35 cycles at 98°C for 7 sec, 55°C for 30 sec, 1 min at 77°C, followed by a final step at 68°C for 5 min. The thermal cycling for *IDH2* was set to 94°C for 30 sec, followed by 35 cycles of 7 sec at 98°C, 30 sec at 58°C, 1 min at 77°C and a final step at 68°C for 5 min. The PCR products were analyzed by electrophoresis, purified, and sequenced. Mutated and wild-type plasmids for *IDH1* and *IDH2* were used to check the accuracy of our analyses. The mutated plasmids harbored the mutations *IDH1R132* and *IDH2R172* for *IDH1* and *IDH2*, respectively. Serial dilutions up to 10% of mutated/wild-type plasmids were analyzed using the same protocol, giving informative results for all dilutions. *IDH1* mutation was spotted even at the lowest concentration of the mutated plasmid (10%), whereas the *IDH2* analysis showed that the mutation could be identified only at concentration >20%.

#### TERT

We amplified the *TERT* promoter region with PCR in order to detect the possible mutations −C228T and −C250T which correspond to positions 124 and 146 nt upstream of the *TERT* ATG start site (7), respectively. DNA was amplified in 25 *μ*l PCR volume containing 1X PrimeSTAR GXL buffer (Takara bio), 200 *μ*M of each dNTP, 0.4 *μ*M of each of the primers, TERTpromF2 and the reverse primer TRETpromR2, 1.25 units of PrimeSTAR GXL DNA polymerase and 20 ng of genomic DNA. The PCR program started with an initial denaturation at 94°C for 30 sec, followed by 35 cycles of 7 sec at 98°C, 30 sec and 90 sec at 68°C and a final extension for 5 min at 68°C. The PCR products were analyzed by electrophoresis, purified and sequenced.

#### Methylation-specific PCR (MSP)

Methylation analysis of the *MGMT* promoter was performed using the primers and PCR conditions described by Esteller *et al* (8) (Table II).

#### Immunohistochemistry

Formalin-fixed paraffin-embedded sections from 23 samples were analyzed for protein expression of HMGA2 using the FLEX+ system (DakoA/S, Glostrup, Denmark). The procedures and the scoring approach were as reported by Hetland *et al* (10).

## Results

### Results of the gene analyses

A complete overview of the results for all the gene analyses is given in Table I.

### HMGA1 and HMGA2 expression

**Figure 1:**
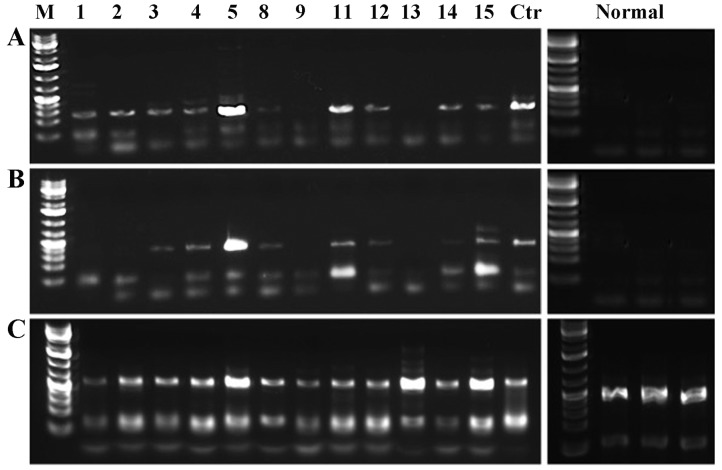
*HMGA2* and *HMGA1* expression detected in vulva tumors and normal controls. (A) RT-PCR using primers for the exon 1–3 region of *HMGA2* for 12 tumors and three normal vulva tissues. Lane M, 1 kb Plus DNA ladder (GeneRuler, Fermentas); lane Ctr, positive control. (B) RT-PCR amplification using primers for exon 1–5 region of *HMGA2*. (C) RT-PCR for *HMGA1*.

**Figure 2:**
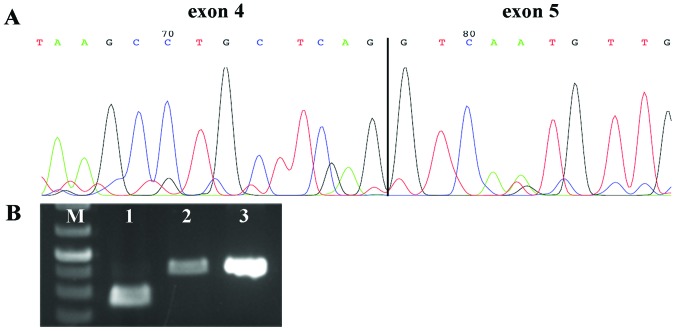
(A) Chromatogram of the *HMGA2* variant 3 found in cases 1 and 2 showing the junction between exon 4 and exon 5. (B) *HMGA2* RT-PCR analyses for case 13. Lane M, 1 kb Plus DNA ladder (GeneRuler, Fermentas); lane 1, RT-PCR using primers for exon 1–3 region of *HMGA2*; lane 2, RT-PCR using primers for exon 1–5 region of *HMGA2*; lane 3: *ABL1* PCR used as the control.

The 12 tumors from which RNA was available were tested for expression of *HMGA1* and *HMGA2* giving informative results for all samples (Table I). The 3 samples from the normal vulva tissue used as controls showed no expression of *HMGA2* but expression of *HMGA1* (Fig. 1). The *HMGA1* gene was expressed both in the 12 tumors and in the controls. For the *HMGA2* gene, the samples were run for two parallel PCR reactions which amplified exons 1–3 and exons 1–5, respectively. Eight cases showed expression of *HMGA2*. Cases 1 and 2 showed expression of a truncated *HMGA2*, i.e., exons 1–3. 3′-RACE PCR was performed in search of possible fusion transcripts in these two cases, and analysis of the sequences revealed the presence in both of them of transcript variant 3 of *HMGA2* (accession no. NM_001300918.1) (Fig. 2A). The *HMGA2* variant 3 contains alternative 3′ coding region and 3′ UTR compared to the normal transcript; this explains why the transcripts found in case 1 and 2 could not be amplified by our primers. The HMGA2 protein expression status was further investigated by immunohistochemistry in the 23 tumors from which material was available (Table I). Four tumors (cases 8, 18, 22 and 23) were negative for HMGA2 immunostaining, but in all other cases HMGA2 expression was noted. Cases 8 and 13 showed contrasting/opposite results with the two methods used, RT-PCR analysis and immunohistochemistry. More specifically, case 8 was found negative for expression of HMGA2 by immunohistochemistry but positive by RT-PCR, whereas case 13 was found positive by immunohistochemistry but negative by RT-PCR. An additional RT-PCR analysis for the latter case was performed following the same protocol, but using a higher concentration of cDNA (30 ng instead of 10 ng) since the immunohistochemistry was positive but with a low score (1–10). We then observed that *HMGA2* was also expressed in case 13 in its entire length (Fig. 2B). The opposite results obtained in case 8 may be due to different part of the biopsy being used for molecular analyses and/or immunohistochemistry.

### TERT

All of the 24 cases from which DNA was extracted were analyzed for mutations in the promoter region of *TERT*. Five tumors (cases 7, 8 and 10–12) showed the C228T. Case 5 showed a C254T unclassified variant.

### IDH1 and IDH2 mutation

Twenty-four samples were analyzed for mutations in *IDH1* and *IDH2*. More precisely, the following mutation sites were investigated: IDH1R100, IDH1R109 and IDH1R132 of *IDH1* and IDH2R140, IDH2R149 and IDH2R172 of *IDH2*. All gave informative results whereas only one sample, case 4 was positive for the SNPI DH1G105.

### MGMT

We assessed *MGMT* promoter methylation using MSP of the 24 samples from which DNA was extracted. All of the tumors gave informative results; however, only one tumor, case 7, was found to have *MGMT* promoter methylation.

## Discussion

**Figure 3:**
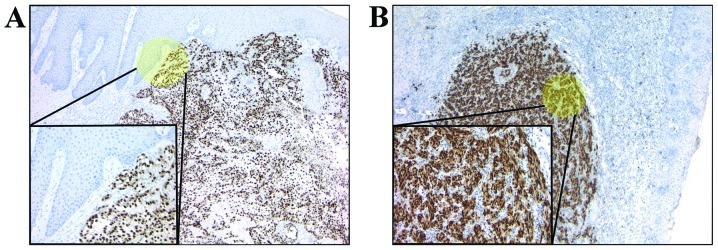
Immunohistochemistry of HMGA2 in vulva tumors. (A) HMGA2 immunostaining of case 5, an SCC (x50, magnification). (B) HMGA2 immunostaining of case 20, a vulvar MM (x50, magnification). Inset shows immunohistochemistry at a higher magnification (x200). SCC, squamous cell carcinoma; MM, malignant melanoma.

The high-mobilty group AT-hook proteins are non-histone proteins involved in a wide variety of nuclear processes from chromatin dynamics to gene regulation; there are two proteins belonging to this group, HMGA1 and HMGA2. The HMGA family genes are expressed during embryonic development (11) but are largely unexpressed in adult normal tissues (12). However, high expression levels of *HMGA2* have been noted in different benign tumors such as lipomas (9), pleiomorphic adenomas of the salivary gland (13), uterine leiomyomas (14) and lung hamartomas (15). In these tumors, *HMGA2* was found disrupted due to rearrangement of chromosome arm 12q. The alterations involve exon 3 and cause deletion of downstream regions leading to a truncated transcript that can evade gene silencing. Alternatively, chromosomal rearrangement of 12q13–15 may lead to formation of a fusion gene. In order to detect a truncated transcript of *HMGA2*, if present, we used two sets of primers for parallel amplification of our samples, one for exons 1–3 and one for exons 1–5. We found a truncated gene in cases 1 and 2 leading to the expression of exons 1–3. We further characterized these transcripts by 3′RACE-PCR searching for possible fusion partners. The karyotypic data on both tumors were normal, so possibly a cryptic rearrangement involving chromosome 12 may be present; alternatively, the cells carrying the chromosomal aberration of interest did not divide *in vitro*. Sequence analysis of the transcripts showed the *HMGA2* splicing variant 3 in both cases. Moreover, immunohistochemistry analysis revealed that HMGA2 was expressed in the majority of tumors, SCC as well as MM (Fig. 3).

This is the first time that expression of the *HMGA2* gene has been assessed in tumors of the vulva. The finding that the entire transcript is expressed in 83% of the samples (including the two cases with the variant form of the gene) give a hint that the gene may be involved in tumorigenesis or tumor progression. Expression of *HMGA2* has hitherto mostly been noted in benign tumors with only sparse or anecdotal information on expression in malignant ones (16). The finding of 20 out of 24 malignant vulvar tumors, both SCC and MM, showing *HMGA2* expression therefore was unexpected. Unfortunately, we did not have sufficient material to investigate for *HMGA2* expression in the only premalignant lesion, the AH, of the present series.

*TERT* is the gene for the telomerase reverse transcriptase. Its involvement in cancer is well known and many studies have shown that mutations in the promoter region can increase telomerase expression (7). We focused on the most frequent mutations, i.e., C228T and C250T, which have been noted in a large number of tumors of different types (17). These mutations introduce a new binding site (TTCCGG) for members of the E-twenty-six/ternary complex factor (Ets/TCF) transcription factor family (17). The C228T mutation was found in 5 of the 6 cases with mutation (out of 24 tumors analyzed). Despite the low frequency of the *TERT* mutation in tumors of the vulva, 25%, it may be important in a subset of SCC. Notably, a single nucleotide variant, C254T, was identified in case 5. This variant/mutation has not been previously reported. Its location is close to the C228T mutation; however, it is not involved in the formation of a new binding site for Ets/TCF. Unfortunately, we did not have material from either normal tissue or blood from this patient and so we could not establish whether the variant was a germ line polymorphism, a rare normal trait or a new cancer-associated mutation.

The *IDH1* and *IDH2* genes express two forms of isocitrate dehydrogenase. Mutations in one of these genes can lead to an enzyme that produces 2-hydroxyglutarate. This metabolite is an inhibitor of α-ketoglutarate-dependent oxygenases, whose impaired activity can cause genome-wide methylations that have an impact on the expression of various genes. Mutations in *IDH1* and/or *IDH2* have been identified in gliomas (6) as well as in hematological malignancies (18). Our analyses showed no mutations of these genes in vulva tumors. SNP IDH1G105, an adverse prognostic factor in CN-AML (19), was present in only a single tumor (case 4). *IDH1* and *IDH2* are probably not involved in vulvar tumorigenesis.

*MGMT* encodes O^6^-methylguanine DNA methyltranferase, a DNA repair enzyme that removes alkyl adducts from the O^6^-position of guanine. Expression of this gene can lead to resistance against alkylating cytostatics. Methylation of the *MGMT* promoter makes the cells more sensitive to alkylating drugs as was demonstrated in different type of cancer, not least gliomas (20). Due to its efficacy as a prognostic and predictive tumor marker, the assessment of *MGMT* promoter methylation status has become one of the most requested analyses for gliomas (21). Our analysis found this gene altered in only a single sample, suggesting that methylation of the *MGMT* promoter of is not a frequent event in vulvar tumorigenesis.

## Figures and Tables

**Table I:** Overview of the results for the vulva tumors.

Case/lab no.	Diagnosis	IDH1	IDH2	TERT	MGMT methylated	HMGA1	HMGA2 ex1–3	HMGA2 ex1–5	Immunohisto-chemistry score
1/02-167	SCC	−	−	−	−	+	+	−	>50
2/02-848	SCC	−	−	−	−	+	+	−	1–10
3/02-869	SCC	−	−	−	−	+	+	+	>50
4/02-1060	SCC	IDH1G105	−	−	−	+	+	+	11–50
5/02-1171	SCC	−	−	C254T	−	+	+	+	>50
6/03-48	AH	−	−	−	−	NA	NA	NA	NA
7/03-830	SCC	−	−	C228T	+	NA	NA	NA	>50
8/03-1011	SCC	−	−	C228T	−	+	+	+	−
9/03-1088	SCC	−	−	−	−	+	−	−	NA
10/04-1190	SCC	−	−	C228T	−	NA	NA	NA	>50
11/06-19	SCC	−	−	C228T	−	+	+	+	1–10
12/06-125	SCC	−	−	C228T	−	+	+	+	11–50
13/06-709	SCC	−	−	−	−	+	+	+	1–10
14/09-733	SCC	−	−	−	−	+	+	+	11–50
15/09-818	SCC	NA	NA	NA	NA	+	+	+	1–10
16/68-98	SCCIS	−	−	−	−	NA	NA	NA	>50
17/02-99	MM	−	−	−	−	NA	NA	NA	1–10
18/00-647	SCC	−	−	−	−	NA	NA	NA	−
19/00-651	SCC	−	−	−	−	NA	NA	NA	>50
20/00-1127	MM	−	−	−	−	NA	NA	NA	>50
21/01-61	SCC	−	−	−	−	NA	NA	NA	>50
22/01-99	SCC	−	−	−	−	NA	NA	NA	−
23/01-134	SCC	−	−	−	−	NA	NA	NA	−
24/01-777	SCC	−	−	−	−	NA	NA	NA	11–50
25/01-981	SCC	−	−	−	−	NA	NA	NA	1–10

SCC, squamous cell carcinoma; SCCIS, squamous cell carcinoma *in situ*; MM, malignant melanoma; AH, atypical squamous cell hyperplasia; NA, not available.

**Table II:** Primers used in the PCR reactions.

Primer name	Primer sequence
IDH1-rs1-86F	5′-CTCCTGATGAGAAGAGGGTTGAG-3′
IDH1-rs1-321R	5′-ACACATACAAGTTGGAAATTTCTGG-3′
IDH2-rs12-42F	5′-CTTGGGGTTCAAATTCTGGTTGA-3′
IDH2-rs12-315R	5′-GCTAGGCGAGGAGCTCCAGTC-3′
TERT-PromF2	5′-GCCGGGCTCCCAGTGGATTCG-3′
TERT-PromR2	5′-GGCTTCCCACGTGCGCAGCAG-3′
HMGA2-846F1	5′-CCACTTCAGCCCAGGGACAACCT-3′
HMGA2-982-F1	5′-CAAGAGTCCCTCTAAAGCAGCTCA-3′
HMGA2-1021R1	5′-CCTCTTGGCCGTTTTTCTCCAGTG-3′
HMGA2-1112R1	5′-CCTCTTCGGCAGACTCTTGTGAGGA-3′
HMGA1-284F1	5′-CAGCCATCACTCTTCCACCTGC-3′
HMGA1-648R1	5′-CTGTCCAGTCCCAGAAGGAAGCT-3′
ABL1-91F1	5′-CAGCGGCCAGTAGCATCTTGACTTTG-3′
ABL1-404R1	5′-CTCAGCAGATACTCAGCGGCATTGC-3′
A3RNV-RACE	5′-ATCGTTGAGACTCGTACCAGCAGAGTCACGAGAGAGACTACACGGTACTGGTTTTTTTTTTTTTTT-3′
A3R1New	5′-TCGTTGAGACTCGTACCAGCAGAGTCAC-3′
A3R3	5′-CGAGAGAGACTACACGGTACTGGT-3′
MSP-MGMT-MetF	5′-TTTCGACGTTCGTAGGTTTTCGC-3′
MSP-MGMT-MetR	5′-GCACTCTTCCGAAAACGAAACG-3′
MSP-MGMT-UnmetF	5′-TTTGTGTTTTGATGTTTGTAGGTTTTTGT-3′
MSP-MGMT-UnmetR	5′AACTCCACACTCTTCCAAAAACAAAACA3′
